# Cytopathological findings of proliferating pilomatricoma misdiagnosed as a malignant parotid gland tumor

**DOI:** 10.1186/s13000-018-0738-4

**Published:** 2018-08-28

**Authors:** Nozomu Kurose, Manabu Yamashita, Mariko Nakano, Xin Guo, Akihiro Shioya, Satoko Nakada, Hiroshi Minato, Sohsuke Yamada

**Affiliations:** 1 Department of Pathology and Laboratory Medicine, Kanazawa Medical University, 1-1 Daigaku, Uchinada, Ishikawa 920-0293 Japan; 2 Department of Pathology, Kanazawa Medical University, Kanazawa, Ishikawa Japan; 3 Department of Diagnostic Pathology, Ishikawa Prefectural Central Hospital, Kanazawa, Ishikawa Japan

**Keywords:** Proliferating pilomatricoma, Basophilic cells, Shadow cells, Cytology

## Abstract

**Background:**

Pilomatricoma is a relatively common benign cutaneous adnexal neoplasm with differentiation towards the hair matrix, inner sheath of hair follicle and hair cortex. Proliferating pilomatricoma is a rare variant of pilomatricoma that can rapidly increase and may be misidentified as a malignant tumor. We herein report the cytopathological findings of proliferating pilomatricoma misdiagnosed as a malignant parotid tumor.

**Case presentation:**

A 64-year-old man noticed an acne-like nodule in the left parotid region. It was painless, but it increased to a maximum diameter of 4.5 cm over 2 years. Clinically, left parotid gland carcinoma was suspected, and fine-needle aspiration cytology was performed. Clusters of epithelial cells were observed in a necrotic background, and malignant epithelial cells derived from salivary glands were suspected. Histologically, the resected tumor was diagnosed as proliferating pilomatricoma composed of basophilic cells and shadow cells apart from the parotid gland. However, on a re-evaluation of the cytological specimens, the irregular-shaped epithelial cells were considered to be from basophilic cells. Shadow cells with nuclear disappearance were also confirmed. Tumor recurrence and metastasis have not been observed in the four years since surgery.

**Conclusion:**

The present case was first interpreted as a malignant parotid gland tumor, but it was actually a benign skin appendage tumor. Pilomatricoma sometimes rapidly increases and may be mistaken for a malignant tumor. Although it is critical to recognize not only basophilic cells but also shadow cells, it cannot be diagnosed by cytological findings. The final diagnosis should be made on excision specimen only.

## Background

Pilomatricoma, also known as calcifying epithelioma, is a relatively common benign cutaneous adnexal neoplasm with differentiation towards the hair matrix, inner sheath of the hair follicle and hair cortex. Pilomatricoma is a hard-nodular lesion that occurs in the head and neck region or upper extremities of children and young adults [1]. It forms an asymptomatic solitary mass and shows a slow-growing course. Histologically, pilomatricoma forms a well-circumscribed nodule and localizes from the dermis to subcutaneous fat tissue. Tumor cells consist of hair matrix-like cells (matrical or basophilic cells) that are basophilic-stained and shadow cells (ghost cells) that are eosinophilic-stained with nuclear concentration and disappearance [1].

Proliferating pilomatricoma is a rare variant of pilomatricoma. It is relatively large, rapidly growing and occurs in the elderly [2]. The subtype is primarily composed of basophilic cells and a small amount of shadow cells, although it can mainly comprise shadow cells in some cases [2].

Parotid region pilomatricoma may be misdiagnosed on fine-needle aspiration (FNA) as primary malignant cutaneous tumor, salivary gland-type malignant tumor or metastatic tumor [3–7]. We herein report a rare case of proliferating pilomatricoma and discuss its cytopathological findings.

## Case presentation

**Fig. 1:**
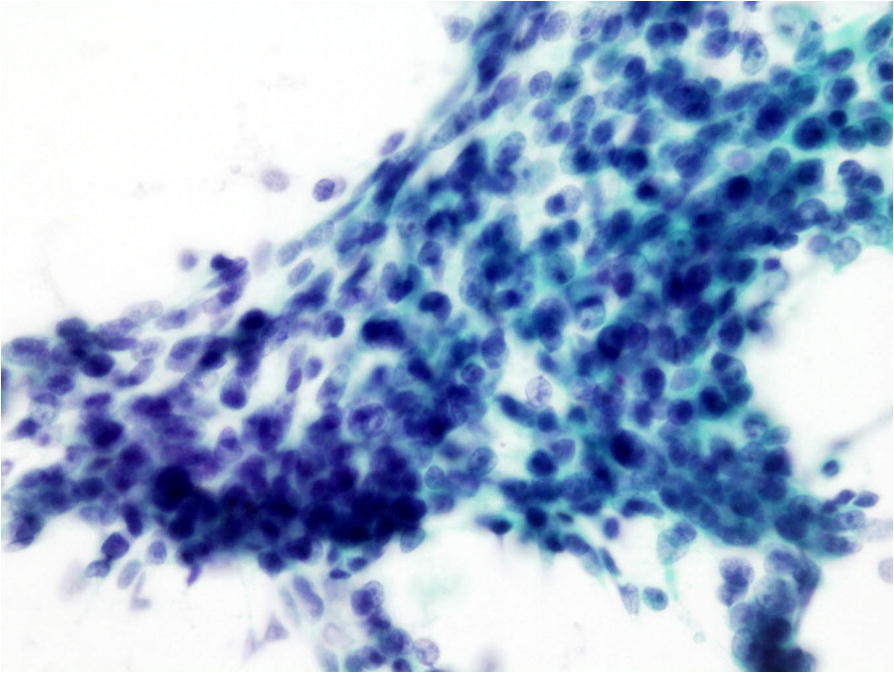
Fine-needle aspiration (FNA) cytology of the left parotid region. A cluster of epithelial cells was observed (Papanicolaou staining, × 400)

**Fig. 2:**
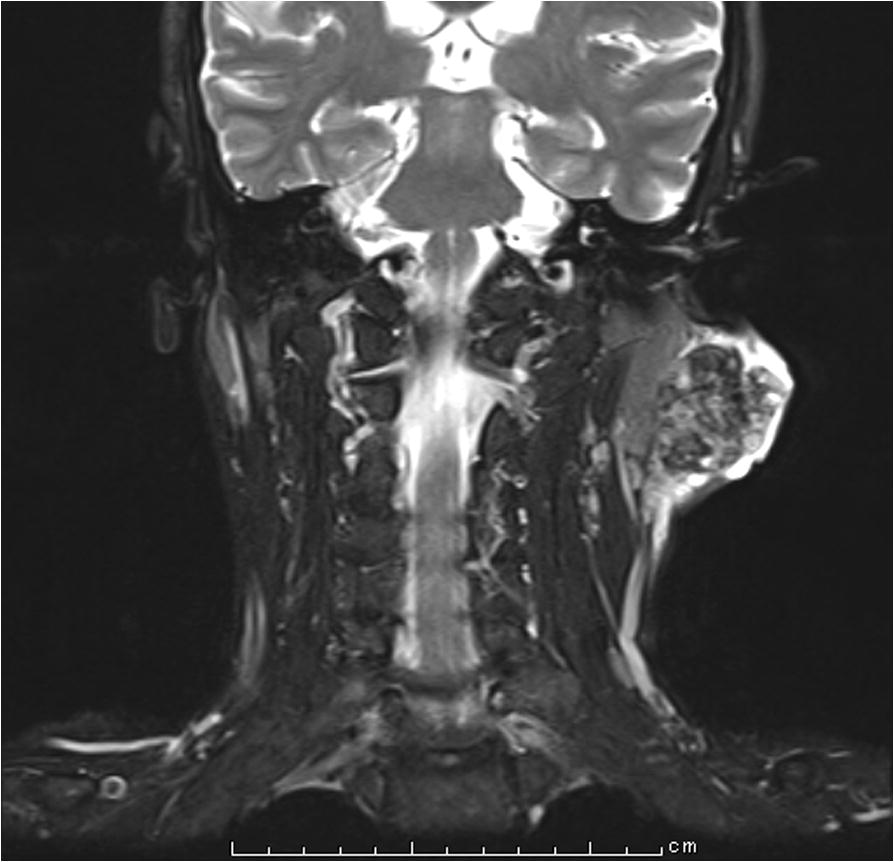
T1-weighted magnetic resonance imaging (MRI) of the left parotid region. A well-defined multilocular tumor with heterogeneous enhancement was seen. The tumor was located close to the outside of the left parotid gland

A 64-year-old man noticed an acne-like nodule in the left parotid region 2 years prior to this presentation. It was painless, but it increased up to a maximum diameter of 4.5 cm. Clinically, left parotid gland carcinoma was suspected, and FNA cytology was performed from the left parotid region. Clusters of epithelial cells were observed in a necrotic and hemorrhagic background. These cell clusters had a sheet-like arrangement and high nuclear-cytoplasmic ratio. The nuclear shape was ovoid with hyperchromasia. Neither nuclear membrane thickening nor irregular-shaped nuclei were noted. One obvious nucleolus was observed in the central portion of the cytoplasm (Fig. 1). Small lymphocytes, histiocytes and multinucleated giant cells were also seen. Malignant epithelial cells derived from salivary glands, including squamous cell carcinoma, myoepithelial carcinoma and carcinoma ex pleomorphic adenoma, were suspected. Magnetic resonance imaging (MRI) revealed a well-defined multilocular tumor located close to the outside of the left parotid gland. On T1- and T2-weighted imaging, low-intensity and heterogeneous gadolinium enhancement was seen (Fig. 2). Radiologically, parotid gland cancer was suspected.

**Fig. 3:**
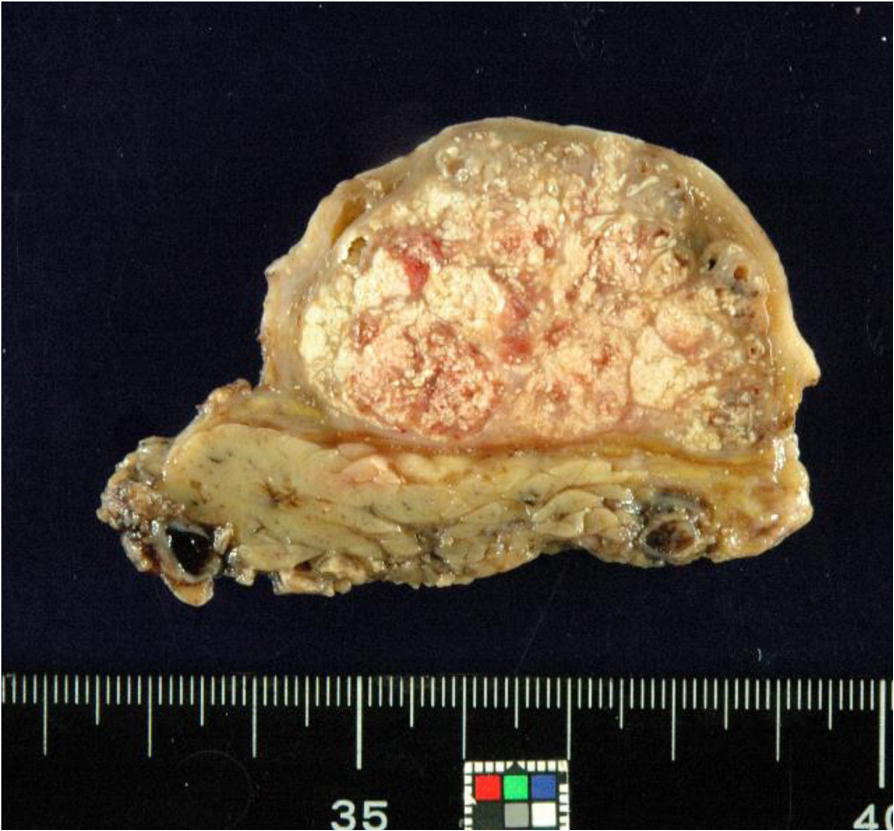
Gross findings of the resected tumor. The cut surface showed a well-defined lobulated tumor containing yellowish, muddy materials

**Fig. 4:**
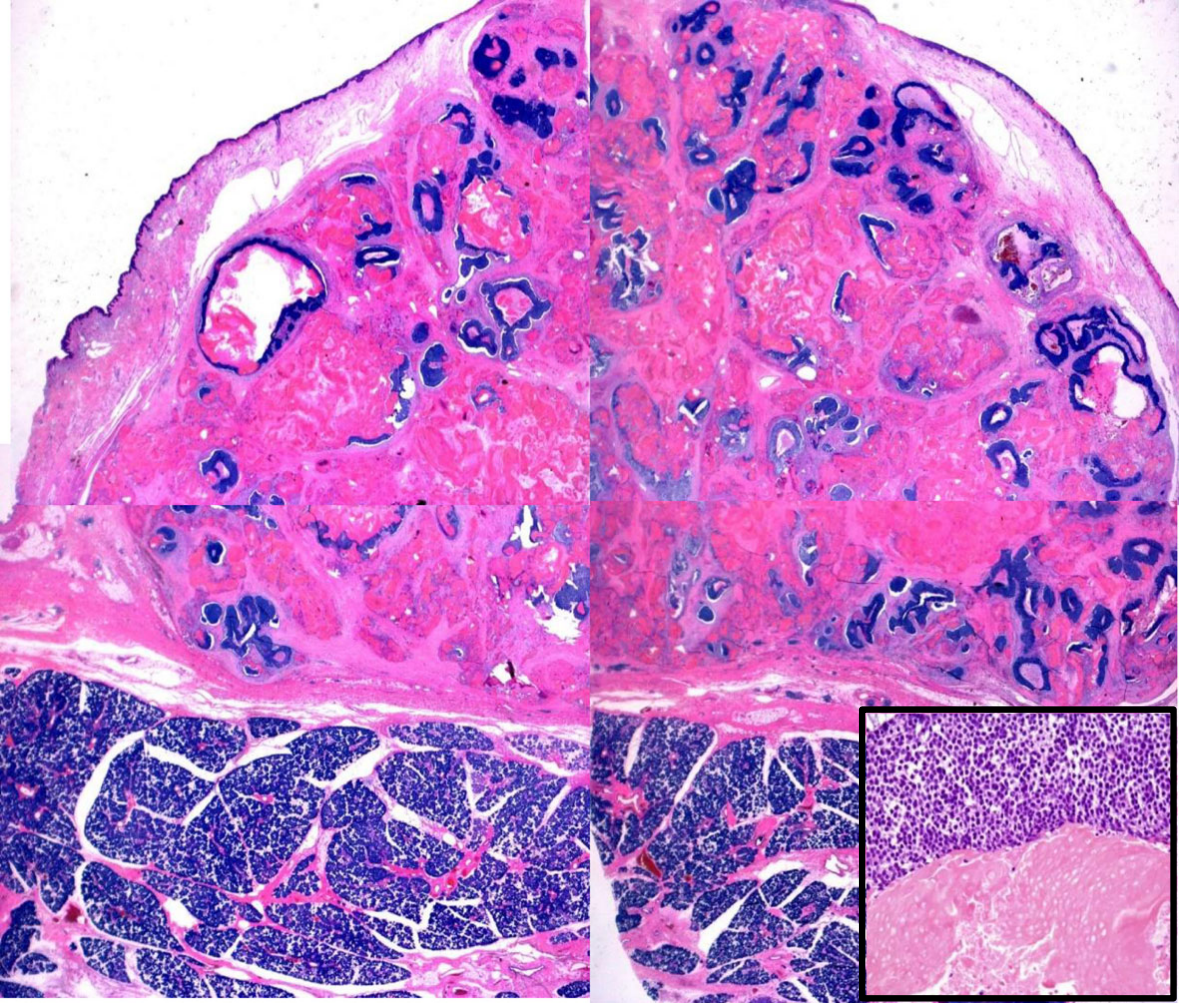
Histological findings of the tumor. The resected tumor cells consisted of shadow cells, and basophilic cells were confirmed at the periphery of the tumor. A normal parotid gland was located in the lower portion (H&E staining, scanning view). Basophilic cells and shadow cells (inset) (H&E staining, × 400)

One month later, tumor resection of the left parotid region and superficial parotidectomy were performed. The cut surface showed a well-defined lobulated tumor containing yellowish-muddy materials (Fig. 3). Histologically, the resected tumor was diagnosed as proliferating pilomatricoma composed of basophilic cells and shadow cells apart from the left parotid gland. The tumor was encapsulated by fibrous tissue without stromal invasion. Approximately 60% of the tumor cells consisted of shadow cells, and basophilic cells were confirmed at the periphery of the tumor. The basophilic cells were oval-shaped with a high nuclear cytoplasm ratio and had an obvious nucleolus. Two mitoses were observed per high-powered field. Focal squamous metaplasia, coagulative necrosis and apoptotic cells were also observed. Eosinophilic-stained shadow cells showed nuclear concentration and disappearance. Transitional histological findings were identified between basophilic cells and shadow cells, and supramatrical cells characterized by incomplete nuclear disappearance were also seen (Fig. 4).

Immunohistochemically, basophilic cells and shadow cells were negative for anti-pan cytokeratin antibody (AE1/AE3, diluted 1:800; Leica) and high-molecular-weight keratin (34βE12, diluted 1:200; DAKO), but squamous metaplastic cells were positive. β-catenin (3-caten, diluted 1:400; DAKO) was positive for basophilic cells with nuclear and cytoplasmic staining. Ki-67 (MIB-1, diluted 1:30; Biogenex) labeling index for basophilic cells and shadow cells were 46.2% and 0%, respectively, and the p53 (Bp53–12, diluted 1:200; IBL) labeling index were 94.8% and 0%, respectively. S-100 protein (2A10, diluted 1:400; IBL), HMB-45 (HMB-45, diluted 1:200; DACO) and Ber-EP4 (Ber-EP4, diluted 1:400; DAKO) were negative for basophilic cells and shadow cells. Fibrosis, calcification, foreign body granulomatous reaction, foamy macrophage aggregation, and lymphocyte infiltration were observed in the tumor stroma. The tumor was completely resected. There was no metastasis to the lymph nodes around the parotid gland.

**Fig. 5:**
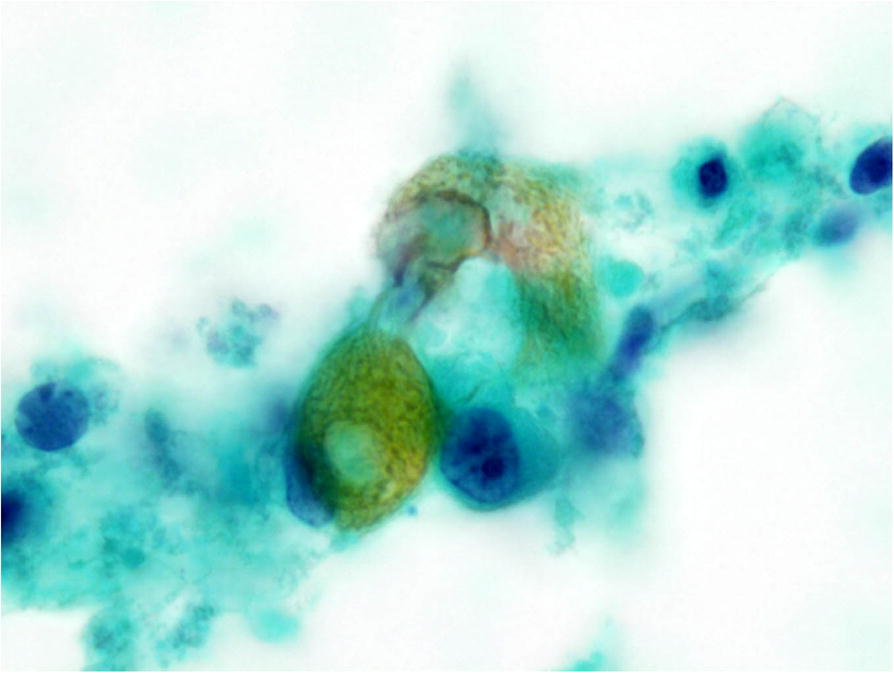
Fine-needle aspiration (FNA) cytology of the left parotid region. Shadow cells with nuclear disappearance were found. Keratin fibers were seen in the cytoplasm of the shadow cells (Papanicolaou staining, × 1,000)

On a re-evaluation of the cytological specimens, the ovoid-shaped epithelial cells were considered to be basophilic cells. Shadow cells with nuclear disappearance were also confirmed. Keratin fibers were found in the cytoplasm of the shadow cells (Fig. 5). Ultimately, we concluded that these cytological findings were consistent with pilomatricoma.

## Discussion

This case report describes a rare proliferating pilomatricoma occurring in the parotid region of an elderly man and rapidly increasing over a period of two years. In addition, the cytological findings of this case were easily misdiagnosed as a malignant tumor, which should be recognized as a diagnostic pitfall.

Proliferating pilomatricoma was a rare variant of pilomatricoma and was first reported by Kaddu et al. in 1997 [2]. According to their English literature, proliferating pilomatricoma occurs often in the head and neck region of the elderly. It rapidly forms a large nodule and rarely ulcerates. Although malignancy is often suspected, it grows symmetrically with pushing margins and lacks vascular or lymphatic permeation and lymph node metastasis [2]. Histologically, this variant is composed mainly of basophilic cells and a small amount of shadow cells but in some cases comprises mainly shadow cells [2]. Furthermore, in the late-phase lesion of pilomatricoma, the growth ability is arrested by the decrease in basophilic cells and the relative increase in shadow cells [8]. Our case showed similar clinicopathological findings to a previous paper, except for the small amounts of basophilic cell and lack of ulceration. Since the present tumor was considered to be a late-phase lesion of completely resected proliferating pilomatricoma, a good prognosis is expected.

On FNA cytology, the presence of basophilic cells causes pilomatricoma to be misdiagnosed as a malignant tumor. Various diseases, including primary malignant skin tumor [7] (e.g. poorly differentiated carcinoma and Merkel cell carcinoma), primary malignant salivary gland tumor [3] (e.g. mucoepidermoid carcinoma), metastatic tumor [6, 7] and small round cell sarcoma [4] (e.g. rhabdomyosarcoma), must be considered, but it is almost impossible to identify pilomatricoma based on the cytological findings of basophilic cells alone. Therefore, it is extremely important to make note of the existence of shadow cells to ensure the accurate cytological diagnosis of pilomatricoma. We did not doubt the clinical diagnosis for parotid gland tumor and failed to properly account for the presence of shadow cells. Cytopathologists must be aware of the diagnostic pitfalls associated with this tumor.

## Conclusions

We herein reported the cytopathological findings of proliferating pilomatricoma. This lesion sometimes rapidly increases and may be mistaken for a malignant tumor. It is critical to recognize not only basophilic cells but also shadow cells. However, it cannot be diagnosed by cytological findings. The final diagnosis should be made on excision specimen only.

## Abbreviations

FNA: Fine-needle aspiration
MRI: Magnetic resonance imaging
